# Association Between Appendectomy and Fibrosis Progression in Nonalcoholic Fatty Liver Disease

**DOI:** 10.4021/gr513w

**Published:** 2013-03-09

**Authors:** Masakazu Nakano, Toshimitsu Murohisa, Yasuo Imai, Masaya Tamano, Hideyuki Hiraishi

**Affiliations:** a Department of Gastroenterology, Dokkyo Medical University, 880 Kitakobayashi, Mibu-machi, Simotsuga-gun, Tochigi, Japan; b Department of Pathology, Dokkyo Medical University Koshigaya Hospital, 2-1-50 Minamikoshigaya, Koshigaya, Saitama, Japan; c Department of Gastroenterology, Dokkyo Medical University Koshigaya Hospital, 2-1-50 Minamikoshigaya, Koshigaya, Saitama, Japan

**Keywords:** Nonalcoholic fatty liver disease, Appendectomy, Fibrosis

## Abstract

**Background:**

A two-hit theory explaining the progression of nonalcoholic fatty liver disease (NAFLD) to nonalcoholic steatohepatitis (NASH) and fibrosis is widely accepted. Endotoxins entering the portal vein from the gut are thought to be one cause of this second hit, and the literature frequently mentions associations between gut-derived endotoxins and progression of fibrosis in NAFLD. The appendix regulates intestinal immunity to protect the gut from the invasion of bacteria and antigens. Appendectomy may thus contribute to progression of fibrosis in NAFLD, but this association has not yet been clarified. We therefore investigated the association between appendectomy and progression of fibrosis in NAFLD.

**Methods:**

Fifty two patients with NAFLD who underwent liver biopsy in our department were included in this study. Based on Brunt’s scores, patients with NAFLD were classified into a mild fibrosis group and advanced fibrosis group.

**Results:**

History of appendectomy was found to be significantly more frequent in patients with advanced fibrosis than in patients with mild fibrosis (P = 0.014). Multivariate logistic analysis was conducted with age, sex, albumin, platelet count, steatosis grade, and history of appendectomy as covariates and advanced fibrosis as the dependent variable. Significant differences were identified for platelet count and history of appendectomy, identifying these as independent risk factors for advanced fibrosis in NAFLD patients. The odds ratio for appendectomy history was 39.415 (P = 0.044).

**Conclusions:**

History of appendectomy was significantly more frequent in NAFLD patients with advanced fibrosis, suggesting that appendectomy may represent a risk factor for advanced fibrosis in NAFLD.

## Introduction

Nonalcoholic fatty liver disease (NAFLD) is the most common chronic liver disease not only in Western industrialized countries, but also in countries of the Asia-Pacific region [1, 2]. With the increase in obesity and diabetes, the prevalence of NAFLD has grown to become a major problem worldwide [3]. Histologically, NAFLD resembles alcoholic liver disease (ALD), but is not attributable to alcohol ingestion. The condition is defined primarily as a liver disease characterized by fatty depositions in the liver and covers a broad spectrum from simple hepatic steatosis to steatohepatitis and hepatic cirrhosis [4, 5]. Within the NAFLD spectrum, simple hepatic steatosis normally does not progress and carries a good prognosis, but nonalcoholic steatohepatitis (NASH) is known to progress to hepatic cirrhosis and hepatocellular carcinoma (HCC) as hepatic fibrosis proceeds [3, 6-8]. The disease state of NAFLD is truly diverse, but progression of hepatic fibrosis is the most important prognostic factor and is of great clinical relevance [9].

A two-hit theory explaining the progression of NAFLD to NASH and fibrosis is widely accepted. This theory attributes pathogenesis to triglyceride deposition in hepatocytes as the first hit, followed by the resulting hepatocyte damage and genetic factors as the second hit. One proposed second-hit mechanism is exposure to gut-derived endotoxin [10]. This is based on two sets of findings: 1) identification of gut-derived endotoxin as a possible prerequisite cofactor in the development of alcoholic steatohepatitis and cirrhosis in alcoholics [11, 12]; and more directly, 2) evidence for the role of gut-derived endotoxin in the pathogenesis of NASH from animal studies [13, 14]. As in ALD, gut-derived endotoxin (lipopolysaccharide (LPS)) in NAFLD travels via the portal circulation to the liver, where it activates the Kupffer cells via toll-like receptor 4 (TLR-4). This in turn induces more tumor necrosis factor-alpha (TNFα) expression and increases levels of reactive oxygen species, causing inflammation and fibrosis in the liver. This contribution of gut-related factors to the progression of NAFLD has been suggested in many reports [13, 15-19].

The appendix is a highly vascular organ that embryologically arises from the cecum and represents an important part of the gut-associated lymphoid tissue system (GALT), together with Peyer’s patches and tonsils. However, for a long period this structure was considered as an evolutionary redundancy that served little, if any, purpose in humans. GALT is actually an important component underlying B-lymphocyte-mediated immune responses and mucosal immune function by extrathymically derived T lymphocytes [20].

Although gut-derived endotoxin is thought to be a factor in NAFLD progression, no research has clarified the relationship of the appendix, which plays a role in mucosal immune function, and the progression of NAFLD to fibrosis. To address this deficit, we decided to investigate the relationship of appendectomy to progression of fibrosis in NAFLD.

## Materials and Methods

### Patients

A retrospective analysis of patient characteristics, clinical data and histopathological information was performed on patients diagnosed with NAFLD or chronic hepatitis B (CHB). This study included NAFLD and CHB patients who underwent liver biopsy at our department between March 1991 and March 2011. A diagnosis of NAFLD was made when the following 3 criteria were met: 1) alcohol consumption ≤ 20 g/day of ethanol; 2) hepatitis B surface antigen and anti-hepatitis C virus antibody-negative status and no distinct evidence of viral hepatic disease or chronic hepatic disease of autoimmune origin; and 3) ≥ 5% fat deposition in hepatocytes as determined from histopathological examination of the liver. Controls comprised patients with CHB, which, unlike NAFLD and chronic hepatitis C patients, is not associated with fatty liver disease [21]. Those of the 1522 patients who proved positive for HBs antigen for ≥ 6 months and had an alcohol consumption ≤ 20 g/day of ethanol were included in the CHB group.

### Clinical and laboratory evaluation

Venous blood samples were taken in the morning after a 12-h overnight fast. All patients underwent measurement of the following laboratory parameters: aspartate aminotransferase (AST); alanine aminotransferase (ALT); γ-glutamyl transpeptidase (γ-GTP); albumin; total cholesterol; triglyceride; fasting plasma glucose; platelet count, and prothrombin time.

A diagnosis of hyperlipidemia was made for patients with total cholesterol ≥ 220 mg/dL, triglycerides ≥ 150 mg/dL or receiving treatment for hyperlipidemia. In accordance with the diagnostic criteria of the Japanese Society of Hypertension, a diagnosis of hypertension was made for patients with systolic blood pressure ≥ 140 mmHg, diastolic blood pressure ≥ 90 mmHg or receiving treatment for hypertension. Based on the diagnostic criteria of the Japan Diabetes Society (JDS), a diagnosis of diabetes mellitus was made for patients with: 1) fasting blood glucose ≥ 126 mg/dL; 2) a 2-h post-load value ≥ 200 mg/dL after a 75-g oral glucose tolerance test; 3) casual blood glucose ≥ 200 mg/dL; or 4) hemoglobin (Hb)A1c (JDS value) ≥ 6.1%, reproducibly, or receiving treatment for diabetes mellitus. Obesity was defined as body mass index (BMI) > 25 kg/m^2^, according to the criteria of the Japanese Obesity Association.

### Pathology

Liver biopsy specimens were stained using hematoxylin-eosin and Azan-Mallory stains and histologically examined by one experienced pathologist who was blinded to the clinical condition and biochemical data of the patient. In NAFLD patients, severity of fibrosis was scored according to the methods of Brunt. Severity of fibrosis for NAFLD patients was expressed on a 4-point scale, as follows: stage 0, normal connective tissue; stage 1, perivenular and/or perisinusoidal fibrosis in zone 3; stage 2, combined pericellular portal fibrosis; stage 3, septal/bridging fibrosis; and stage 4, cirrhosis. On the basis of this classification, subjects were grouped into two categories by the fibrosis: those with mild fibrosis (stage 0 - 2); and those with advanced fibrosis (stage 3 - 4) in NAFLD patients. In CHB patients, severity of fibrosis was scored according to the methods of Knodell. Severity of fibrosis for CHB patients was expressed on a 4-point scale, as follows: score 0, no fibrosis; score 1, fibrous portal expansion; score 3, bridging fibrosis (portal-portal or portal-central linkage); score 4, cirrhosis. On the basis of this classification, subjects were grouped into two categories by the fibrosis: those with mild fibrosis (score 0 - 1); and those with advanced fibrosis (score 3 - 4) in CHB patients according to NAFLD group. Degree of steatosis was assessed based on the percentage of hepatocytes containing macrovesicular fat droplets, as follows: grade 0, no steatosis; grade 1, < 33% of hepatocytes containing macrovesicular fat droplets; grade 2, 33-66% of hepatocytes containing macrovesicular fat droplets; and grade 3, > 66% of hepatocytes containing macrovesicular fat droplets. Severity of lobular inflammation was expressed as follows: none, no foci; mild, < 2 foci per ´ 200 field; moderate, 2 - 4 foci per ´ 200 field; and severe, > 4 foci per ´ 200 field by NAFLD Activity Score [22].

### Statistical analysis

Comparison of clinical and histological features between groups was performed across fibrosis. Results are expressed as the median (range), or the number (percentage) of patients with each variable. Univariate analysis was conducted using the Mann-Whitney test to assess the significance of between-group differences in continuous variables. The χ^2^ test was used to compare frequency data. Multivariate logistic regression analysis was used to analyze independent factors related to fibrosis progression. SPSS version 11.0 statistical software (SPSS, Chicago IL, USA) was used for data analysis, and values of P < 0.05 were judged as significant. The study protocol was approved by the ethical committee of Dokkyo Medical University. All subjects gave informed written consents.

## Results

Fifty-two NAFLD patients and 68 CHB patients who underwent liver biopsy met the predefined diagnostic criteria. Histological data are summarized in Table 1. Twenty-eight of the 52 NAFLD patients were stage 0 - 1, 12 were stage 2, 8 were stage 3, and 4 was stage 4. Forty patients were in the mild fibrosis group (stages 0 - 2) and 12 were in the advanced fibrosis group (stages 3 - 4). Seventeen patients (32.7%) showed steatosis grade 1, 23 (44.2%) showed steatosis grade 2, and 12 (23.1%) showed steatosis grade 3. Thirty-three of the 68 CHB patients showed advanced fibrosis with bridging fibrosis corresponding to Brunt stage 3.

**Table 1:** Histological Features of Patients With NAFLD and CHB

	NAFLD patientsn = 52	CHB patientsn = 68
Brunt fibrosis stage		
0-1	28 (53.8%)	
2	12 (23.1%)	
3	8 (15.4%)	
4	4 (7.7%)	
Knodell fibrosis score		
0		9 (13.2%)
1		26 (38.2%)
3		26 (38.2%)
4		7 (10.9%)
Mild fibrosis	40	35
Advanced fibrosis	12	33
Severity of Steatosis		
0	0 (0%)	37 (54.4%)
1	17 (32.7%)	21 (30.9%)
2	23 (44.2%)	9 (13.2%)
3	12 (23.1%)	1 (1.5%)

A summary of the hematology test results and physical data for the 52 NAFLD and 68 CHB patients were presented in Table 2. In NAFLD patients, the male-to-female ratio was 31:21, and median age was 48.0 years. Median BMI was high, at 27.7 kg/m^2^, and 75% of all patients were found to be obese. Twenty-four patients (46.2%) had diabetes mellitus, 35 (67.3%) had hyperlipidemia, and 32 (61.5%) had hypertension. Nineteen of the NAFLD patients overall (36.5%) and 12 of the control group CHB patients overall (17.6%) had a history of appendectomy. History of appendectomy tended to be more frequent among NAFLD patients than among CHB patients (P = 0.020). The NAFLD patient was a result than CHB patient with many mergers of metabolic factor such as obesity, diabetes, hyperlipidemia and hypertension. The histological data show that NAFLD patients displayed significantly higher grade of steatosis than CHB patients.

**Table 2:** Clinical Features of NAFLD and CHB Patients, and Univariate Comparison of Clinical and Histological Features of Patients

	NAFLD patients	CHB patients	P value
Number	52	68	
Age	48	39.5	0.102
Gender (Female)	21 (40.4%)	22 (32.4%)	0.365
BMI (kg/m^2^)	27.7	22.8	< 0.001
Obesity (BMI > 25)	39 (75.0%)	21 (30.9%)	< 0.001
Diabetes	24 (46.2%)	5 (7.4%)	< 0.001
Hyperlipidemia	35 (67.3%)	22 (32.4%)	< 0.001
Hypertension	32 (61.5%)	22 (32.4%)	0.002
AST (U/L)	63	49.5	0.319
ALT (U/L)	92.5	78.5	0.136
Total bilirubin (mg/dL)	0.6	0.7	0.134
Albumin (g/dL)	4.2	4	< 0.001
Triglycerides (mg/dL)	151	95	< 0.001
Total cholesterol (mg/dL)	207	177	< 0.001
Glucose (mg/dL)	101	89	< 0.001
Platelet count (10^4^/µL)	19.8	16.1	0.004
Prothrombin time (%)	100	95.5	0.115
Appendectomy	19 (36.5%)	12 (17.6%)	0.020
Steatosis (grade 0/1/2/3)	0/23/17/12	37/21/9/1	0.001

Clinical and laboratory data of NAFLD and CHB patients for the two fibrosis groups are shown in Table 3. History of appendectomy was significantly more frequent among NAFLD patients with advanced fibrosis than among NAFLD patients with mild fibrosis (P = 0.014). NAFLD Patients with advanced fibrosis were significantly older than those with mild fibrosis (P = 0.002), and were significantly more often female (P = 0.006). The following liver function parameters were worse in NAFLD patients with advanced fibrosis: albumin (P = 0.016); platelet count (P < 0.001); and prothrombin time (P < 0.001). The histological data show that patients with advanced fibrosis displayed significantly lower grade of steatosis (P = 0.036). In CHB patients, the following liver function parameters were worse with advanced fibrosis: platelet count (P < 0.004); and prothrombin time (P < 0.003). On the other hand, history of appendectomy was not difference between advanced fibrosis and mild fibrosis in CHB patients.

**Table 3:** Univariate Comparison of Clinical and Histological Features Between the Two Groups of NAFLD or CHB Patients Divided by Degree of Fibrosis

	NAFLD			CHB		
	Mild fibrosis	Advanced fibrosis	P value	Mild fibrosis	Advanced fibrosis	P value
Number	40	12		35	33	
Age	40	59.0	0.002	37	41	0.390
Gender (Female)	12 (30.0%)	9 (75.0%)	0.006	11 (31.4%)	11 (33.3%)	0.868
BMI (kg/m^2^)	27.7	28.0	0.983	21.8	23.8	0.052
Obesity (BMI > 25)	29 (72.5%)	10 (83.3%)	0.452	8 (22.9%)	13 (39.4%)	0.143
Diabetes	16 (40.0%)	8 (66.7%)	0.108	2 (5.7)	3 (9.1%)	0.597
Hyperlipidemia	28 (70.0%)	7 (58.3%)	0.454	12 (34.3%)	10 (30.3%)	0.728
Hypertension	24 (60.0%)	8 (66.7%)	0.680	10 (28.6%)	12 (36.4%)	0.496
AST (U/L)	55	73.5	0.487	50	49	0.750
ALT (U/L)	93	81.5	0.307	76	79	0.589
Total bilirubin (mg/dL)	0.6	0.8	0.081	0.8	0.6	0.083
Albumin (g/dL)	4.3	4.0	0.016	4	3.7	0.160
Triglycerides (mg/dL)	156.5	128.0	0.400	82	105	0.393
Total cholesterol (mg/dL)	209	193	0.196	169	178	0.599
Glucose (mg/dL)	96	111	0.424	85	92	0.233
Platelet count (10^4^/µL)	22.8	14.2	< 0.001	18.5	15.2	0.004
Prothrombin time (%)	100	77.5	< 0.001	98.6	86	0.003
Appendectomy	11 (27.5%)	8 (66.7%)	0.014	6 (17.1%)	6 (18.2%)	0.911
Steatosis (grade 0/1/2/3)	0/11/17/12	0/6/6/0	0.036	21/7/6/1	16/14/3/0	0.717

Multivariate logistic analysis was conducted with age, sex, albumin, platelet count, history of appendectomy, and grade of steatosis (which showed significant associations on univariate analysis) as covariates and advanced fibrosis as the dependent variable in NAFLD patients (Table 4). Significant differences were identified for platelet count and history of appendectomy, indicating that these factors represented independent risk factors for advanced fibrosis in NAFLD patients. The odds ratio for appendectomy was 39.42 (95% confidence interval, 1.11 - 1,400.73; P = 0.044).

**Table 4:** Multiple Logistic Regression Analysis of Factors Associated With Advanced Fibrosis in NAFLD Patients

	Odds ratio	95% CI	P value
Age	1.009	0.825 - 1.139	0.500
Gender(Female)	3.805	0.264 - 54.79	0.753
Albumin(g/dL)	0.120	0.001 - 14.45	0.460
Platelet count (10^4^/µL)	0.349	0.137 - 0.891	0.033
Appendectomy	39.415	1.109 - 1400.73	0.044
Steatosis	8.045	0.556 - 116.3	0.103

## Discussion

Overall lifetime risk of appendicitis is 8.6% for males and 6.7% for females. Lifetime risk of appendectomy, which is most often an abdominal surgical emergency, is 12.0% for males and 23.1% for females [23]. Peak incidence of appendectomy occurs in the second and third decades, but appendicitis can occur at any age [23]. History of appendectomy was more frequent among our NAFLD patients (33.3%) than among CHB patients (17.6%). The finding that history of appendectomy was significantly more frequent among NAFLD patients with advanced fibrosis than among NAFLD patients with mild fibrosis indicates that advanced fibrosis represents an independent risk factor in NAFLD patients. In patients with advanced fibrosis, history of appendectomy was significantly more frequent among NAFLD patients than among CHB patients. History of appendectomy thus appears to represent a risk factor for fibrosis progression in NAFLD.

The appendix is thought to have some immune functions based on its association with substantial lymphatic tissue. Based on: a) recent advances in the understanding of immune-mediated biofilm formation by commensal bacteria in the mammalian gut; b) biofilm distribution in the large bowel; c) the association of lymphoid tissue with the appendix; d) the potential for biofilms to protect and support colonization by commensal bacteria; and e) the architecture of the human bowel, the human appendix is believed to be well suited as a “safe house” for commensal bacteria, providing support for bacterial growth and potentially facilitating re-inoculation of the colon in the event that the contents of the intestinal tract are purged following exposure to a pathogen [24]. The intestinal tract represents the port of entry for many microbes. The intestinal tract contains GALT, a specialized concentration of lymphoid tissue that defense against microbial invasion. The appendix, together with the tonsils and Peyer’s patches of the terminal ileum, is a component of GALT. GALT is one of the first defensive barriers against microorganisms and other environmental and food antigens. T and B lymphocytes (plasma cells) function in GALT, mainly segregating immunoglobulin (Ig)A [24-26]. GALT represent a site of B-cell activation, proliferation, and terminal differentiation in response to antigens. The continuous presence of bacterial antigens necessitates dynamic remodeling of GALT and the selection of multi-layered strategies for protection. One important mechanism of protection is achieved through the production of large amounts of secretory IgA (sIgA) by plasma cells residing in the lamina propria (LP) of the gut. These sIgAs are secreted mainly as dimers or larger polymers after incorporation of a J chain and association with a polymeric Ig receptor [27]. GALT is usually divided into two functional compartments, known as inductive and effector sites [27]. The primary inductive sites, where most of the IgA immune responses are initiated, include organized follicular structures present in the gut wall, particularly of the small intestine. The intestinal LP is considered the main mucosal effector site, involved in the final maturation of plasmablasts into plasma cells and in the secretion of sIgA into the gut lumen [28]. The most important role in the GALT system is played by sIgA, which is traditionally thought to guard against bacterial translocation by serving as an immunological defense against the intestinal flora and barrier of the intestinal wall [29].

On the other hand, a two-hit theory explaining the progression of NAFLD to NASH and fibrosis is widely accepted, and gut-derived endotoxin is thought to be a factor of second hit in NAFLD progression. Currently proposed mechanisms for endotoxemia in patients with NASH include: a) bacterial overgrowth; and b) disrupted intestinal barrier integrity (leaky gut) that results in increased absorption of endotoxin [14, 30-32]. Wigg et al studied the association of bacterial overgrowth to the progression of NAFLD by analyzing TNFα levels with the ^14^C-D-xylose-lactulose breath test. Small intestinal bacterial overgrowth was a feature in NASH patients not seen in controls [31]. Li et al administered a probiotic in a mouse model of NAFLD. This treatment corrected small intestinal bacterial overgrowth and improved liver histology and insulin resistance [33].

Integrity of the intestinal barrier influences NAFLD progression in several ways. Fructose, ethanol, starvation, and aspirin increase intestinal permeability, leading to higher levels of gut-derived endotoxin and portal endotoxemia. NAFLD consequently progresses to advanced fibrosis [31, 32, 34-38].

In a recent investigation of the relationship between appendectomy and intestinal immunity, Juan et al. reported that GALTectomy (appendectomy and/or tonsillectomy) significantly decreases sIgA levels in serum. This decrease is more marked when both operations have been performed in the same patient. This decrease continues for between 3 months and 3 years in appendectomized patients and more than 20 years in tonsillectomized patients [39].

By primarily regulating sIgA, the appendix creates an intestinal barrier to protect the intestine. Intestinal defense capacity falls with the decrease in sIgA following appendectomy, likely leading to a rise in gut-derived endotoxin due to bacterial overgrowth and disrupted intestinal barrier integrity. This may cause the progression of NAFLD to fibrosis.

Obesity results in increased hepatotoxicity and decreased survival after exposure to LPS in both of the obese strains of Zucker fatty/fatty rats and obese/obese mice, suggesting two mechanisms that might mediate obesity-related sensitivity to endotoxin: altered Kupffer cell function; and increased hepatocyte sensitivity to TNF-α [13]. Recent studies have shown that obese mice display enhanced intestinal permeability leading to increased portal endotoxemia that makes hepatic stellate cells more sensitive to bacterial endotoxins [14]. History of appendectomy may have been more frequent in our NAFLD patients with advanced fibrosis than in CHB patients because increased endotoxin sensitivity in NAFLD, which is closely associated with obesity and hepatic steatosis, may promote fibrosis progression.

Appendectomy has also been associated with chronic liver disease in primary biliary cirrhosis (PBC). Appendectomy, other abdominal surgeries, and tonsillectomy were significantly more frequently reported in patients with PBC in an epidemiological study in North America [40]. Rigopoulou et al reported a case-control study based on a consecutive and unselected cohort of patients with PBC attending a Liver Unit, in which patients were compared with age- and gender-matched controls with other liver diseases (chronic hepatitis B and C) attending the same unit during the same period of time [41]. The linkage to appendectomy was theoretically attractive, since a PBC-specific immune response to the highly conserved caseinolytic protease P of *Yersinia enterocolitica* in 40% of patients with PBC was reported [42]. Of note, infection with *Y. enterocolitica* is one of the major causes of acute terminal ileitis mimicking acute appendicitis [43].

The association between appendectomy and inflammatory bowel disease (IBD) has been studied at many sites. Many studies have found that appendectomy is uncommon in patients with ulcerative colitis (UC) [44-47]. A case-control study by Naganuma et al found that 6.5% of UC patients had undergone appendectomy, compared to 16.3% of control patients, suggesting that appendectomy may inhibit the pathogenesis of UC. In addition, fewer appendectomized UC patients suffer recurrence compared to their non-appendectomized counterparts [44]. Such findings suggest that alterations in mucosal immune responses leading to appendicitis or resulting from appendectomy may negatively affect the pathogenetic mechanisms underlying UC.

In conclusion, we found that appendectomy was common in NAFLD patients with advanced fibrosis, suggesting history of appendectomy as a risk factor for progression of fibrosis in NAFLD. This research appears to be the first to suggest an association between NAFLD and the appendix. Despite the demonstrated role of appendectomy-induced reductions in sIgA levels, which control intestinal immunity, whether increased endotoxin concentrations in the portal circulation of appendectomized NAFLD patients actually cause fibrosis progression remains unclear. This topic warrants further research. Given the small sample size of our study, the potential association between appendectomy and fibrosis progression in NAFLD needs to be revisited in a prospective study on a larger scale.
